# Comparative study of midterm outcomes between Roux-en-Y gastric bypass (RYGB), diverted one-anastomosis gastric bypass (D-OAGB), and one anastomosis gastric bypass (OAGB)

**DOI:** 10.1007/s00423-024-03525-3

**Published:** 2024-11-09

**Authors:** Mohamed Abdul Moneim El Masry, Islam Abdul Rahman, Mohamed Fathy Mahmoud Elshal, Ahmed Maher Abdul Moneim

**Affiliations:** 1 Faculty of Medicine, Cairo University, Giza, Egypt; 2 Military Production Specialized Medical Centre, Helwan, Egypt

**Keywords:** One anastomosis gastric bypass (OAGB), diverted OAGB, Roux-en-Y gastric bypass (RYGB), Weight loss, Gastroesophageal reflux

## Abstract

**Purpose:**

Diverted one anastomosis gastric bypass (D-OAGB) is a new procedure that entails performing Roux-en-Y diversion during OAGB to preclude post-OAGB bile reflux. This study aimed to compare the mid-term outcomes of Roux-en-Y gastric bypass (RYGB) and OAGB versus D-OAGB.

**Methods:**

This is a retrospective study that encompassed the analysis of data from patients undergoing bypass surgeries from 2015 to May 2021. The patients’ data until 2 years of follow-up were compared.

**Results:**

This study included 140 patients who underwent OAGB (*n* = 64), RYGB (*n* = 24), and D-OAGB (*n* = 52). In the OAGB, RYGB, and D-OAGB groups, complication rates were 3.1%, 8.3%, and 5.8%, respectively. At the 3-month and 6-month follow-ups, the OAGB and D-OAGB groups showed a statistically significant higher percentage of excess weight loss (EWL%). Otherwise, the weight measures and weight loss outcome were comparable among the three groups in the other follow-up visits (*p* > 0.05). There was a significantly lower number of gastroesophageal reflux disease (GERD) remission cases and a higher number of de novo GERD cases in the OAGB group.

**Conclusion:**

D-OAGB demonstrated favorable outcomes, including lower early adverse events and superior weight loss results in the first 6 months post-surgery when compared to RYGB. The D-OAGB group also showed higher rates of GERD remission and lower de novo GERD occurrence than OAGB. Further research is warranted to validate these findings and expand our understanding of this innovative surgical approach.

**Supplementary Information:**

The online version contains supplementary material available at 10.1007/s00423-024-03525-3.

## Introduction

Obesity has become a pandemic, impacting people’s health worldwide [1]. Currently, metabolic/bariatric surgery is the most definitive solution for obesity and its related medical conditions [2]. Sleeve gastrectomy (SG) and Roux-en-Y gastric bypass (RYGB) have been recognized as the gold standard bariatric procedures. However, Roux-en-Y gastric bypass has shown a more evident metabolic effect and remission of obesity-related conditions due to the bypass component of the surgery [3].

One anastomosis gastric bypass (OAGB) is another bypass procedure that was first described by Rutledge and published in 2001 [4]. Since then, it has gained wide popularity and acceptance, being a relatively simple procedure compared to RYGB and maintaining its effectiveness in weight loss and resolution of obesity-related medical conditions. It has even been reported that OAGB has weight loss and metabolic benefits exceeding those of RYGB due to the more malabsorptive effect driven by the longer biliopancreatic limb [5]. One anastomosis gastric bypass has risen to become the third most frequently conducted bariatric surgery, trailing only sleeve gastrectomy (SG) and Roux-en-Y gastric bypass (RYGB) [6]. In addition, it gained recognition as a mainstream bariatric technique by the International Federation for the Surgery of Obesity and Metabolic Disorders (IFSO) in 2018 [7].

However, there is still a controversial OAGB-related clinical concern, which is the biliary reflux that is responsible for surgery revision in a number of patients [8]. Diverted One-Anastomosis Gastric Bypass (D-OAGB) is a new procedure that entails performing Roux-en-Y diversion during OAGB to preclude the post-OAGB bile reflux and associated marginal ulcers seen with OAGB [9]. It has been noted as a less complex approach compared to RYGBP, as it circumvents the necessity of positioning the small bowel up to the gastroesophageal junction, thereby reducing tension on the anastomosis [9]. This study aimed to compare the mid-term outcomes of RYGB and OAGB versus D-OAGB.

## Patients and methods

This is a retrospective study that entailed the analysis of prospectively collected data. The study was conducted on consecutively recruited patients who were planned for bariatric bypass surgery at our institutions during the period from December 2015 to May 2021. The study was initiated after getting the approval of the Research Ethics Committee and adhering to the Declaration of Helsinki.

The patient’s eligibility for bariatric surgery was judged per the IFSO guidelines [10, 11]. Patients recruited for bypass surgeries (OAGB, RYGB, or D-OAGB) during the study period were included in the study. The selection of either procedure was based on the preference of each patient after a discussion with the surgeon that included a dedicated description of each choice’s advantages and possible shortcomings. The study patients underwent routine workups that included detailed history-taking, multidisciplinary clinical evaluation, and laboratory investigations. Patients with incomplete follow-up data until 2 years after surgery were excluded from the study. Written informed consent was obtained from each patient before surgery.

## Operative procedure

The surgical procedures were conducted as established. In summary, the surgery was performed under general anesthesia. In a diamond-shaped pattern, five trocar insertions were done after the induction of pneumoperitoneum.

In patients who underwent OAGB, the gastric pouch was fashioned over a 36-French bougie, and after determining the Treitz ligament, a bilio-pancreatic limb (BPL) of 200 cm length was created. A 3-cm side-to-side gastrojejunal anastomosis was formulated to be vertical or slightly oblique, ante-colic, and isoperistaltic.

In patients who were subjected to the RYGB group, the BPL was created at a length of 45 cm and anastomosed side-to-side with the gastric pouch, and a 120 cm alimentary limb (AL) was performed along the mesenteric border.

In patients undergoing D-OAGB, steps were followed the same as in OAGB. The stomach was vertically divided using a stapling device over a 36-French bougie to create a long and narrow gastric pouch. From the ligament of Treitz, which marks the start of the jejunum, 200 cm of the small intestine is measured. This section was designated as the BPL. An additional segment, measuring 60 cm distally, was marked as the alimentary limb, the end of which was surgically connected to the lower part of the gastric pouch, forming a direct path for the food to bypass a large portion of the stomach and the initial segment of the small intestine. This connection is known as the gastrojejunal anastomosis. One of the distinguishing steps of the D-OAGB is the creation of the entero-enterostomy, a secondary connection in the small intestine. To achieve this, antimesenteric holes are fashioned both in the previously identified BPL and near the terminus of the alimentary limb. These holes allow for the establishment of the secondary connection, diverting the bile and pancreatic fluids to join the food contents further downstream. As a result of the procedure, the intestine takes on a Roux-en-Y configuration. This design ensures that food bypasses the initial section of the intestines, promoting malabsorption while also facilitating the diversion of bile and pancreatic fluids, thus reducing the chance of bile reflux into the stomach. Stapling was used for the anastomosis. Barbed sutures were done using a 3 − 0 polydioxanone 15 cm, 26 mm needle [9, 12]. Diagrams illustrating surgical procedures are shown in supplementary file 1.

In the three groups, an intraoperative methylene blue test was employed to examine for the presence of any anastomotic leakage. Drain usage was determined selectively based on the presence of any bleeding spots and tissue conditions.

### Study outcomes

The primary outcome of this study was the weight loss outcome, evaluated through the percentage of weight loss at 3-month, 6-month, and subsequent follow-ups. Secondary outcomes included perioperative adverse events for each surgical group, GERD outcomes, and the improvement of obesity-related medical conditions.

The percentages of total weight loss (TWL%) and excess body weight loss (EWL%) were calculated as previously described [13]. Improvement of hypertension, diabetes mellitus, and dyslipidemia was considered if one or more medications’ dosage was reduced or cessated [14]. Patients were diagnosed to have GERD if they had typical symptoms and/or were using medical treatment such as proton pump inhibitors (PPIs) after the prescribed 3-month postoperative use, according to the described guidelines [15]. GERD improvement was considered in cases of symptom relief or cessation of treatment.

### Statistical analysis

The patients’ data were analyzed using version 28 of the SPSS statistical software (IBM Corp., Armonk, NY, USA). After ensuring data normality using the Shapiro-Wilk test, the numerical data were expressed as mean and standard deviation (SD), and the categorical data were displayed as count and percentage. Numerical data of the three groups were compared using the ANOVA test. A post hoc test (Tukey’s test) was used to assess the statistical significance between each two groups. Qualitative data were presented as counts and percentages, and the chi-square test was used for comparison. A P-value less than 0.05 was considered statistically significant.

## Results

**Fig. 1:**
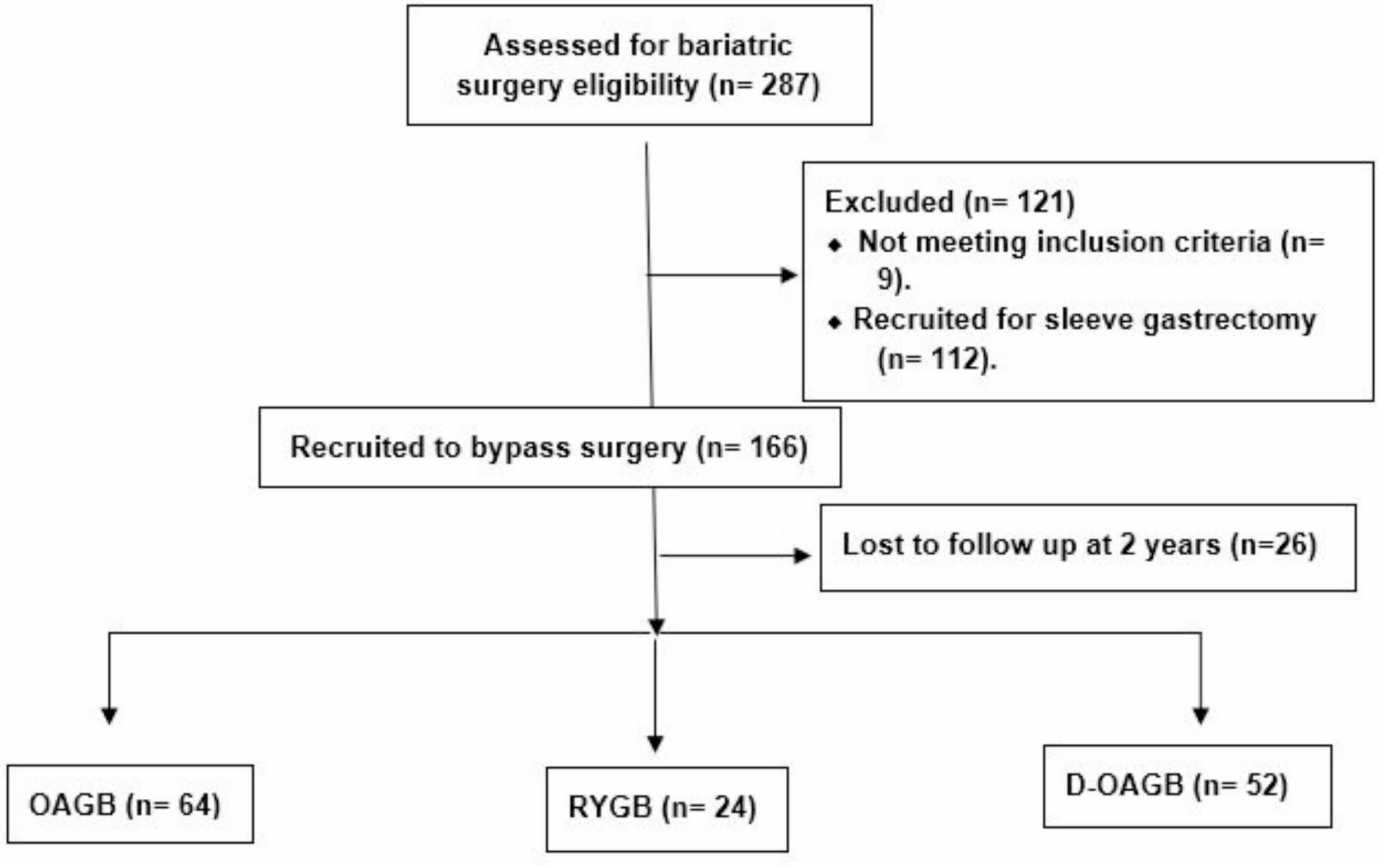
Patients’ flow chart

The patients’ main obesity-related medical conditions were hypertension (*n* = 39, 27.9%), diabetes mellitus (*n* = 42, 30.0%), dyslipidemia (*n* = 68, 48.6%), and non-alcoholic fatty liver disease (NAFLD: *n* = 89, 63.6%). Gastroesophageal reflux symptoms were found in 34 patients (24.3%). The patients in the three groups were comparable in the baseline criteria, apart from the GERD rate, which was significantly lower in the OAGB group (Table 1).

**Table 1:** Baseline demographic data of the study patients

		The OAGB group (*n* = 64)	The RYGB group (*n* = 24)	The D-OAGB group (*n* = 52)	*p*-value
		Mean ± SD	Mean ± SD	Mean ± SD	
Age (year)		37.5 ± 10.3	35.3 ± 10.2	39.04 ± 9.2	0.304
Preoperative weight (Kg)		124.97 ± 23.96	120.83 ± 17.69	123.08 ± 23.04	0.735
Preoperative BMI (Kg/m^**2**^)		45.1 ± 6.8	46.5 ± 8.7	43.8 ± 7.3	0.330
		**Count (%)**	**Count (%)**	**Count (%)**	
Sex	Male	13 (20.3)	5 (20.8)	19 (36.5)	0.114
	Female	51 (79.7)	19 (79.2)	33 (63.5)	
**Obesity-related medical conditions**					
Hypertension		17 (26.6)	9 (37.5)	13 (25)	0.29
Type 2 diabetes mellitus		18 (28.1)	9 (37.5)	15 (28.8)	0.468
Dyslipidemia		28 (43.8)	13 (54.2)	27 (51.9)	0.568
NAFLD		39 (60.9)	15 (62.5)	35 (67.3)	0.517
GERD		7 (1.1)	9 (37.5)	18 (34.6)	0.003*

*: statistically significant

### Operative and early postoperative events

A lower mean surgery time was found in the OAGB group. However, without statistical significance (110.47 ± 29.33 min in the OAGB group, 121.04 ± 36.71 min in the RYGB group, and 119.9 ± 33.95 min in the D-OAGB group, *p* = 0.206), no intraoperative adverse events were experienced. Early postoperative complications occurred in 7 patients (5.0%), and surgical intervention was required in 4 of them (2.9%). In the OAGB group, complications were recorded in 2 patients (3.1%), including one case of septicemia managed conservatively and one case of intra-abdominal bleeding necessitating exploration. The RYGB group exhibited a higher complication rate at 8.3% (*n* = 2), with two cases of leakage that required re-intervention and stent placement. In the D-OAGB group, complications were noted in 3 cases (5.8%), consisting of one case of intra-abdominal bleeding necessitating exploration and drainage, another bleeding case managed conservatively, and one leakage case treated conservatively (Table 2).

**Table 2:** Operative and early postoperative events and weight loss outcomes

Parameter		OAGB (*n* = 64)	RYGB (*n* = 24)	D-OAGB (*n* = 52)	*p*-value
Mean Surgery Time (mins)		110.47 ± 29.33	121.04 ± 36.71	119.9 ± 33.95	0.206
Early Postoperative Complications (%)		2 (3.1%)	2 (8.3%)	3 (5.8%)	0.528
BMI (kg/m^2)	3 month	34.2 ± 6.25	37.15 ± 5.84	33.76 ± 4.98	0.049*
	Post-hoc	p1 = 0.048*, p2 = 0.681, p3 = 0.011*			
	6 month	31.9 ± 4.2	33.3 ± 4.5	30.5 ± 5.7	0.346
	1 year	29.7 ± 47	32.8 ± 4.3	29.4 ± 4	0.088
	2 year	27.02 ± 5.1	28.4 ± 5	26.9 ± 4	0.670
EWL%	3 month	43.68 ± 8.15	37.21 ± 11.85	42.4 ± 9.34	0.016*
	Post-hoc	p1 = 0.005*, p2 = 0.432, p3 = 0.043*			
	6 month	67.33 ± 9.35	61.4 ± 13.17	66.11 ± 8.15	0.040*
	Post-hoc	p1 = 0.021*, p2 = 0.461, p3 = 0.031*			
	1 year	79.92 ± 17.8	67.83 ± 11.7	81.94 ± 22.1	0.084
	2 year	93.01 ± 18.8	87.83 ± 21.8	94.08 ± 35.9	0.805
TWL%	3 month	17.7 ± 5.5	17.4 ± 5.9	18.9 ± 4.4	0.427
	6 month	26 ± 8.9	24.1 ± 6.7	26.3 ± 4.5	0.365
	1 year	31.7 ± 7.9	31.5 ± 5.6	34 ± 7.6	0.385
	2 year	33.9 ± 8.7	34.5 ± 6.6	35.6 ± 7.8	0.520
**1-year obesity-related medical conditions remission rate**					
Hypertension		34/39 (87.2%)	7/9 (77.8%)	12/13 (92.3%)	0.596
Diabetes Mellitus		14/18 (77.8%)	6/9 (66.7%)	8/10 (80.0%)	0.743
Dyslipidemia		27/28 (96.4%)	11/13 (84.6%)	25/27 (92.6%)	0.403
NAFLD		14/39 (35.9%)	5/15 (33.3%)	12/35 (34.3%)	0.981

*: statistically significant, p1: p-value for the comparison between the OAGB and RYGB groups, p2: p-value for the comparison between the OAGB and D-OAGB groups, p3: p-value for the comparison between the RYGB and D-OAGB groups

### Weight loss outcome

At the 3-month follow-up, the OAGB and D-OAGB groups showed statistically significant lower weight (93.2 ± 19.19 kg and 92.97 ± 18.67 kg vs. 105.71 ± 18.26 kg in the RYGB group, *p* = 0.033), lower BMI (34.2 ± 6.25 kg/m^2^ and 33.76 ± 4.98 kg/m^2^ vs. 37.15 ± 5.84 kg/m^2^ in the RYGB group, *p* = 0.049), and higher EWL% (43.68 ± 8.15 and 42.4 ± 9.34 vs. 37.21 ± 11.85 in the RYGB group, *p* = 0.016). No statistically significant differences were noted in the TWL% (*p* = 0.427) (Table 2).

At the 6-month follow-up, similar significant differences were noted in the weight (79.5 ± 11.23 kg and 80.2 ± 12.31 kg vs. 86.5 ± 11.81 kg in the RYGB group, *p* = 0.042) and EWL% (67.33 ± 9.35 and 66.11 ± 8.15 vs. 61.4 ± 13.17 in the RYGB group, *p* = 0.040) (Table 2).

Otherwise, the weight measures and weight loss outcome were comparable among the three groups in the other follow-up visits (*p* > 0.05) (Table 2).

### Obesity-related medical conditions

The three groups showed significant improvement in the obesity-related medical conditions (Table). At the 1-year postoperative follow-up, remission occurred in 34/39 patients (87.2%) with hypertension (88.2% of the OAGB group, 92.3% of the D-OAGB group, and 77.8% of the RYGB group, *p* = 0.596), 32/42 patients (76.2%) with diabetes mellitus (77.8% of the OAGB group, 80% of the D-OAGB group, and 66.7% of the RYGB group, *p* = 0.743), 63/68 patients (92.6%) with dyslipidemia (96.4% of the OAGB group, 92.6% of the D-OAGB group, and 84.6% of the RYGB group, *p* = 0.403), and 31/89 patients (34.8%) with NAFLD (35.9% of the OAGB group, 33.3% of the D-OAGB group, and 34.3% of the RYGB group, *p* = 0.981) (Table 2).

At 6 months, relief of symptoms occurred in 23/34 patients (67.6%) with GERD (14.3% of the OAGB group, 83.3% of the D-OAGB group, and 77.8% of the RYGB group, *p* = 0.003), and worsening occurred in 2/34 patients (5.9%), both of whom were in the OAGB. De novo GERD symptoms were experienced by 2 patients (in the OAGB). At the one-year and 2-year follow-ups, the number of denovo cases reached 4 patients (3 in the OAGB group and 1 in the D-OAGB group).The mid-term outcome of the study groups is presented in Table 3 and demonstrated no statistically significant differences among groups.

**Table 3:** The mid-term outcome of the study groups

Outcome	OAGB	RYGB	D-OAGB	*p*-Value
Internal hernias	2 (3.1%)	1 (4.2%)	1 (1.9%)	0.849
Anastomotic ulcers	3 (4.7%)	2 (8.3%)	0 (0.0%)	0.117
Conversions Due to complications	1 (1.6%)	0 (0.0%)	0 (0.0%)	0.751
Suboptimal clinical response	5 (7.8%)	2 (8.3%)	2 (3.8%)	0.654

## Discussion

The very new procedure, diverted one anastomosis gastric bypass, also named long pouch Roux-en-Y bypass (LPRYGB), was performed in 2013 and published in 2018. First, it has been implemented by Ribeiro and coworkers as a revisional procedure for patients who have failed OAGB due to reflux symptoms. After that, the procedure was performed as a primary procedure. Its first name, diverted one anastomosis gastric bypass, has been grounded on being typical of OAGB, with adding a second entero-enterostomy aiming at diversion of the bile away from the stomach to preclude postoperative bile reflux and GERD [9]. The second name, long pouch Roux-en-Y gastric bypass, has been mentioned in their article published in 2019, since they considered the added entero-enterostomy as a Roux-en-Y diversion [12].

The foundation of our understanding and subsequent practice of this procedure was derived from scientific meetings with the surgery innovators during 2014 and 2015, where they extensively discussed and demonstrated the technique. Encouraged by the promise it showed, we began implementing this procedure by the end of 2015.

As for the best of our knowledge, this is the first study reporting practicing this procedure after being introduced by Ribeiro et al. [9, 12]. In our study, for the first time, we compared the outcome of this procedure to the most established bypass surgeries (RYGB and OAGB).

Overall, OAGB and D-OAGB showed outperformance in terms of lower early postoperative adverse events. However, the difference did not reach the level of statistical significance, likely due to the relatively small sample size. This result aligns with the previously reported fewer OAGB-associated adverse events compared to RYGB, which has been attributed to the only anastomosis and the more straightforward anatomical construction found in OAGB [16]. In spite of the presence of a second anastomotic site in the D-OAGB, the surgery doesn’t entail bringing up an intestinal loop up to the gastroesophageal junction, which likely makes up less tension on the site of anastomosis [17]. It is worthy to note that this D-OAGB-associated lower rate of early adverse events, compared to RYGB, comes in the early learning curve of the procedure, with likely lower rates as the learning curve progresses.

The present study showed another point of OAGB and D-OAGB superiority, which was the significantly better weight loss outcome during the first year after surgery. This is consistent with a meta-analysis encompassing 16 studies and 12,445 patients that found that OAGB was associated with a higher postoperative EWL% compared to RYGB [18]. This was explained by the longer BPL in both surgeries compared to that of RYGB.

Notably, a significantly lower number of GERD remissions and a higher number of denovo GERD cases were found in the OAGB group. This is in line with the still-debatable association between OAGB and biliary reflux [19–22]. The bile reflux-associated Barrett’s esophagus and gastric cancer have been described [23, 24], which makes it a non-negligible surgery consequence. For this reason, RYGB has long been regarded as the best therapeutic choice for GERD patients [25–27]. The newly adopted procedure could now be another solution for patients with GERD. It offers efficiency in GERD remission and early weight loss, along with a lower rate of adverse events and denovo GERD occurrence, making it an excellent choice for patients seeking bariatric surgery, especially those with GERD, or risky for GERD and Barrett’s esophagus.

The findings of this study indicate that OAGB, RYGB, and D-OAGB present similar mid-term outcomes, with no statistically significant differences in internal hernias, anastomotic ulcers, or conversions due to complications. The RYGB group showed a nearly double rate of anastomotic ulcers compared to the OAGB group, despite not reaching statistical significance. These findings align with the study of Vitiello et al. [28] that found a non-significantly higher rate of anastomotic ulcer in the RYGB group than the OAGB group. Additionally, internal hernias were reported to occur less frequently after OAGB compared to RYGB [29]. However, these findings challenge previous studies suggesting that OAGB presents a higher risk of mid-term complications when compared with other surgeries such as single anastomosis duodeno-ileal bypass (SADI), as reported by Gallucci et al. [30]. The observed discrepancies could stem from differences in surgical expertise, patient selection, or follow-up protocols.

Overall, our work emphasizes the new procedure-associated promising outcome that was reported by Ribeiro et al. [9, 12]. The study is limited by the small sample size. D-OAGB is a new and non-popular procedure, thus limiting the patient’s acceptability of the procedure. The study is also limited by the retrospective design, short-term assessment, non-objective assessment of GERD, and deficient data regarding the nutritional deficiencies in the included patients. However, we present our preliminary experience in a very new procedure that needs to be unveiled by more comparative studies.

## Conclusion

In conclusion, D-OAGB, also known as LPRYGB, demonstrated favorable outcomes, including lower early adverse events and superior weight loss results in the first year when compared to RYGB, with GERD remission and reduced de novo GERD occurrence when compared to OAGB. Further research is warranted to validate these findings and expand our understanding of this innovative surgical approach.

## Electronic Supplementary Material

Below is the link to the electronic supplementary material.

Supplementary Material 1

## Data Availability

No datasets were generated or analysed during the current study.
