# Supplementary material for: Comparative study of midterm outcomes between Roux-en-Y gastric bypass (RYGB), diverted one-anastomosis gastric bypass (D-OAGB), and one anastomosis gastric bypass (OAGB)
Source: Langenbecks Arch Surg. 2024 Nov 9;409(1):340. doi: 10.1007/s00423-024-03525-3 (PMC11550272; doi:10.1007/s00423-024-03525-3)

| Oesophagogastric junction distance | A | 1 cm |
| --- | --- | --- |
| Bougie | B | 40 F |
| Length of Pouch | C | 15 cm |
| Length of biliopancreatic limb | D | 200 cm |
| Lenth of enteric Roux limb | E | 60 cm |
| Width of Gastrojejunostomy | F | 3 cm |
| Width of Jejunojejunostomy | G | 4.5 cm |
| Common channel | H | As is |


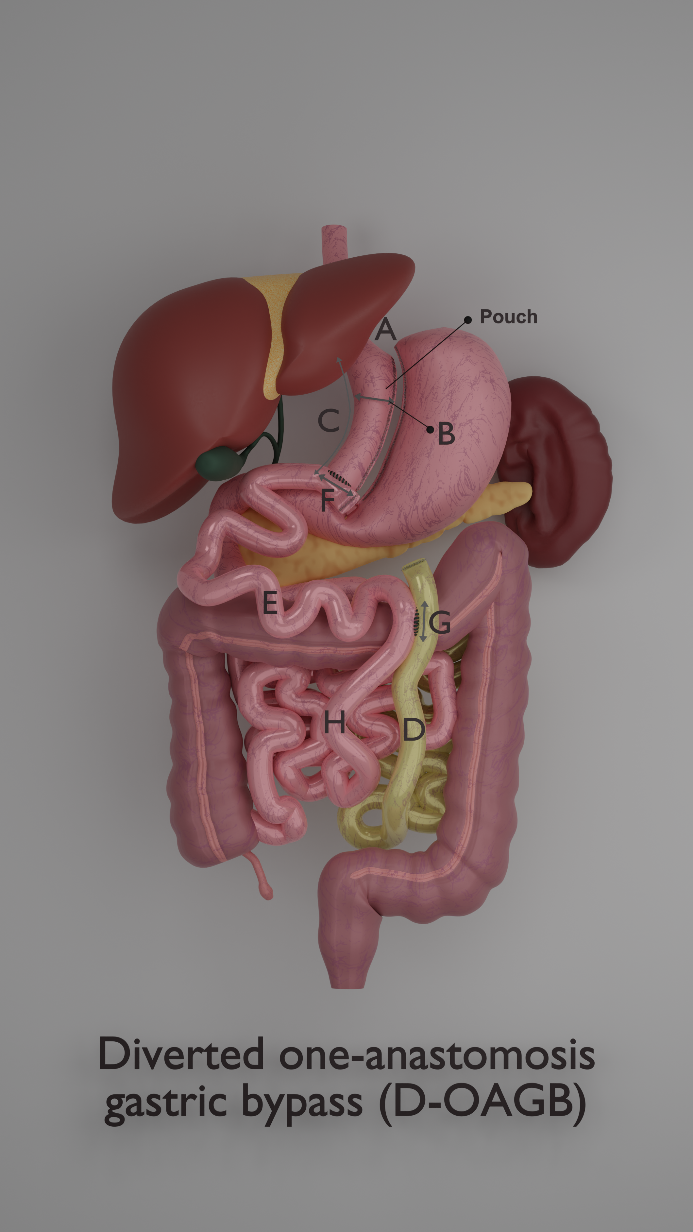


| Oesophagogastric junction distance | A | 1 cm |
| --- | --- | --- |
| Width of Pouch | B | 3 cm |
| Length of Pouch | C | 3 cm |
| Length of enteric Roux limb | D | 120 cm |
| Length of biliopancreatic limb | E | 45 cm |
| Width of Gastrojejunostomy | F | 3 cm |
| Width of Jejunojejunostomy | G | 4.5 cm |


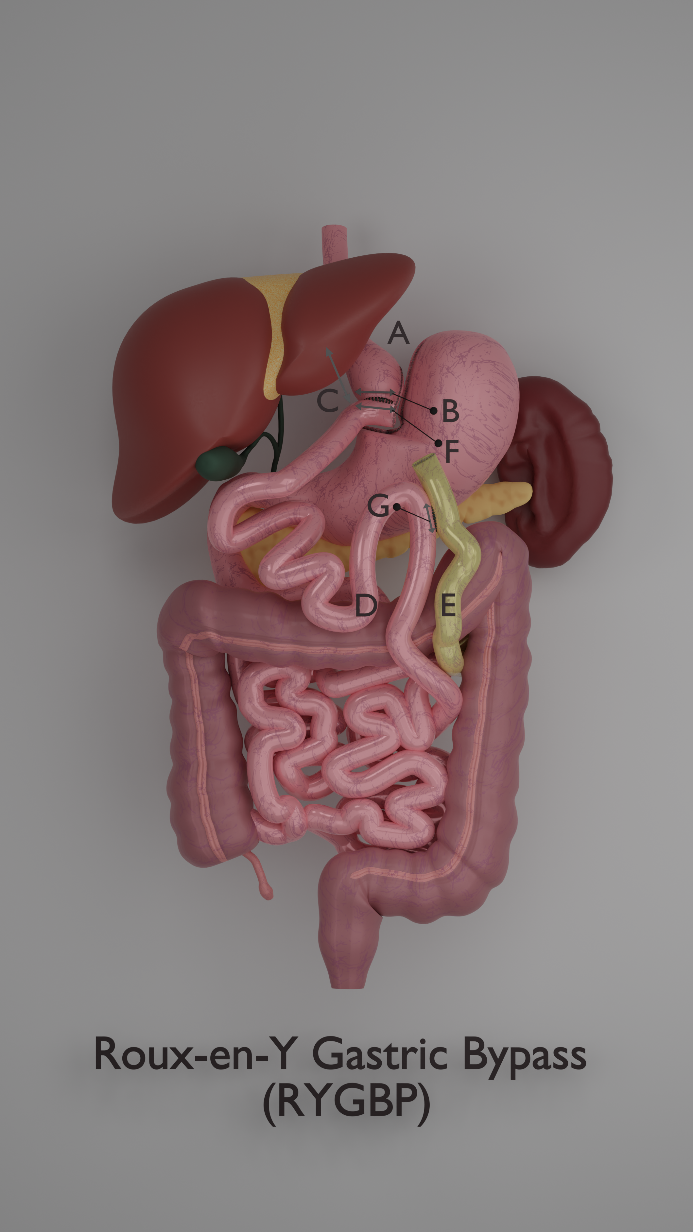


| Oesophagogastric junction distance | A | 1 cm |
| --- | --- | --- |
| Bougie | B | 40 F |
| Length of Pouch | C | 15 cm |
| Length of biliopancreatic limb | D | 200 cm |
| Width of Gastrojejunostomy | E | 3 cm |
| Common channel | F | As is |


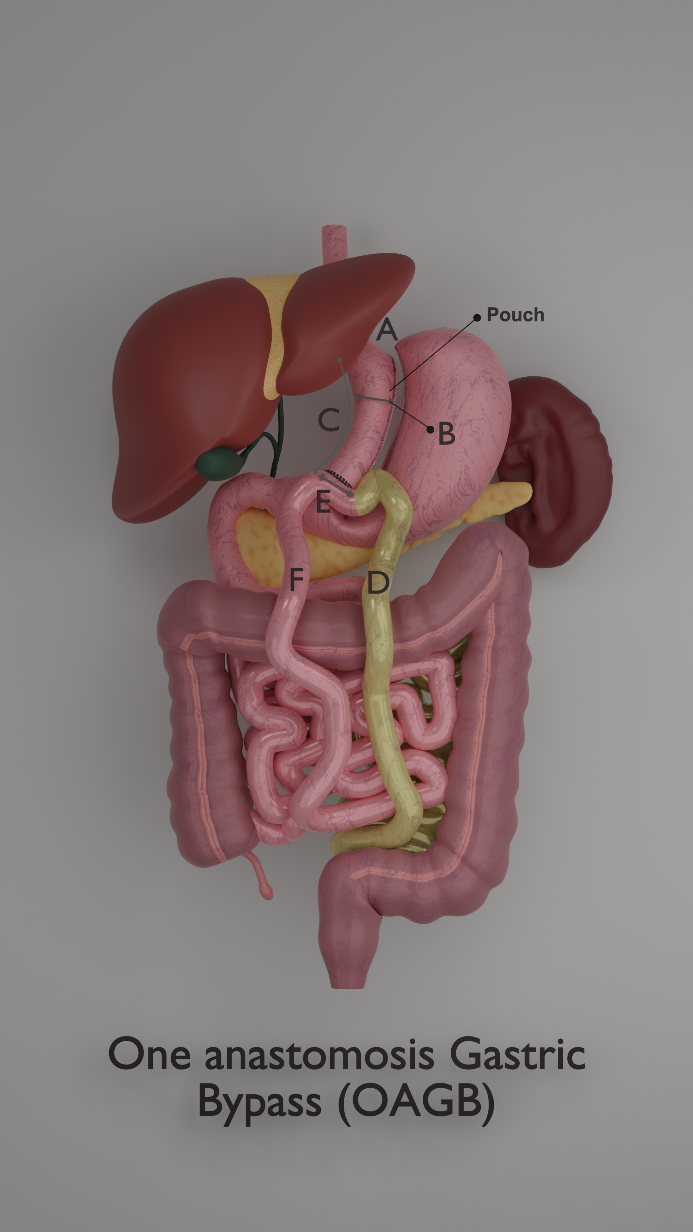

Supplement: Supplementary file 1 — Supplementary Material 1 [file 423_2024_3525_MOESM1_ESM.docx]
